# The current landscape of machine learning-based radiomics in arteriovenous malformations: a systematic review and radiomics quality score assessment

**DOI:** 10.3389/fneur.2024.1398876

**Published:** 2024-06-10

**Authors:** Audrey A. Grossen, Alexander R. Evans, Griffin L. Ernst, Connor C. Behnen, Xiaochun Zhao, Andrew M. Bauer

**Affiliations:** ^1^Department of Neurosurgery, University of Oklahoma Health Sciences Center, Oklahoma City, OK, United States; ^2^Data Science and Analytics, University of Oklahoma, Norman, OK, United States

**Keywords:** arteriovenous malformation, radiomics, radiogenomics, artificial intelligence, machine learning, deep learning, precision medicine

## Abstract

**Background:**

Arteriovenous malformations (AVMs) are rare vascular anomalies involving a disorganization of arteries and veins with no intervening capillaries. In the past 10 years, radiomics and machine learning (ML) models became increasingly popular for analyzing diagnostic medical images. The goal of this review was to provide a comprehensive summary of current radiomic models being employed for the diagnostic, therapeutic, prognostic, and predictive outcomes in AVM management.

**Methods:**

A systematic literature review was conducted according to the Preferred Reporting Items for Systematic Reviews and Meta-Analyses (PRISMA) 2020 guidelines, in which the PubMed and Embase databases were searched using the following terms: (cerebral OR brain OR intracranial OR central nervous system OR spine OR spinal) AND (AVM OR arteriovenous malformation OR arteriovenous malformations) AND (radiomics OR radiogenomics OR machine learning OR artificial intelligence OR deep learning OR computer-aided detection OR computer-aided prediction OR computer-aided treatment decision). A radiomics quality score (RQS) was calculated for all included studies.

**Results:**

Thirteen studies were included, which were all retrospective in nature. Three studies (23%) dealt with AVM diagnosis and grading, 1 study (8%) gauged treatment response, 8 (62%) predicted outcomes, and the last one (8%) addressed prognosis. No radiomics model had undergone external validation. The mean RQS was 15.92 (range: 10–18).

**Conclusion:**

We demonstrated that radiomics is currently being studied in different facets of AVM management. While not ready for clinical use, radiomics is a rapidly emerging field expected to play a significant future role in medical imaging. More prospective studies are warranted to determine the role of radiomics in the diagnosis, prediction of comorbidities, and treatment selection in AVM management.

## Introduction

Arteriovenous malformations (AVMs) are rare vascular anomalies involving dysplastic arteries and veins with no intervening capillaries and little to no brain parenchyma. The annual rupture rate of AVMs has been reported to be as high as 2–4% with re-hemorrhage rates closer to 6–7% (1–7). Current therapies include routine surveillance, endovascular embolization, microsurgery, and stereotactic radiosurgery (SRS). While there are clinical trials underway studying the efficacy of medications in AVMs, their role in management has yet to be fully established (8). Often, multimodality treatment of AVMs is warranted, using a combination of methods.

The management strategy of AVMs is controversial and dependent on a number of factors including patient anatomy, past medical history, and surgeon preference (9, 10). It is difficult to predict behavior of AVMs due to a large amount of hemodynamic heterogeneity (10, 11). A number of grading systems have been used to predict surgical morbidity and most of these are dependent on the imaging and angiographic appearance of the AVM. Treatment decisions are, in large part, made from assessment of multiple imaging modalities; most useful when analyzed in combination. While catheter angiography is diagnostic, other imaging methods such as CT, CTA, MRI, and MRA can provide additional information about adjacent brain that cannot be fully assessed with catheter angiography alone.

In the past decade, medical imaging analysis has grown to be quite sophisticated. Increasing data set sizes and pattern recognition tools have led to the development of systems which allow for the conversion of images into mineable data, and can subsequently be used in clinical decision-making and support (12). The term “radiomics” was first coined in 2012 by Philippe Lambin, a Dutch research and radiation oncologist (13). While the field grew around oncology and tumor management, it has begun to expand to other medical niches. Today, radiomics is understood to be a research field of medical imaging which can utilize artificial intelligence (AI) to quantitatively measure parameters of the visual information on standard medical imaging (12, 14). Machine learning (ML) and deep learning (DL) methods have both been applied toward this goal. Applying algorithms to imaging databases has the potential to lead to the discovery of radiomic features undetectable to the naked eye which can aid in clinical decision-making. The goal of this review was to provide a comprehensive summary of current radiomic models being employed for the diagnostic, therapeutic, prognostic, and predictive outcomes in AVM management. Specifically, we aim to assess the role of radiomics in the clinical course of AVM patients undergoing imaging and/or therapeutic intervention in the context of conventional assessment modalities. Additionally, we describe the workflow of radiomics, quality of included models, current challenges, and future opportunities in precision medicine.

## Methods

We conducted this review in accordance with PRISMA (Preferred Reporting Items for Systematic Reviews and Meta-Analyses) guidelines (15).

### Search strategy

We performed a systematic search of the PubMed and Embase databases to identify studies published up to April 26, 2024 using the terms: (cerebral OR brain OR intracranial OR central nervous system OR spine OR spinal) AND (AVM OR arteriovenous malformation OR arteriovenous malformations) AND (radiomics OR radiogenomics OR machine learning OR artificial intelligence OR deep learning OR computer-aided detection OR computer-aided prediction OR computer-aided treatment decision). The clinicaltrials.gov website was also searched for existing clinical trials.

Articles were screened by title and/or abstract by two independent reviewers based on proper inclusion and exclusion criteria. Reviews, case reports, letters to the editor, commentaries, abstracts published from academic conferences, and articles not accessible in English were excluded. Articles were included if they (1) presented original research, (2) involved imaging of patients diagnosed with AVM (3) employed manual or automatic methods to segment these images and (4) identified and extracted radiomic features to assess in the diagnosis, therapeutic decisions, prognosis, or predictive models of AVM.

### Data extraction and outcomes of interest

Full text review was conducted by the same two reviewers, with the following metrics extracted: study objectives, number of patients with imaging assessed, imaging modalities, scanner, the number of radiomic features included in the model, AI component, and all reported statistics from training and validation models [ROC AUC, Sensitivity, specificity, true positives (TPs), true negatives (TNs), false positives (FPs), and false negatives (FNs), negative predictive value (NPV), and positive predictive value (PPV)]. In cases in which researchers presented data for various models examining the same variable were tested, the performance metrics for the best-performing model were extracted.

### Radiomics quality score

To qualitatively assess each article, we calculated a Radiomics Quality Score (RQS) for each of the included studies following the criteria set forth by Lambin et al. (16). In short, 16 variables were extracted from each study and awarded a certain amount of points according to the outlined criteria (16). The maximum score was 36. If the information for a certain variable was not stated within the article, the study received no points or deductions for that particular variable.

### Performance metrics definitions

*Training:* First stage of algorithm development, in which model weights and biases are adjusted during continuous passes through a dataset to minimize error.

*Testing:* Second stage of algorithm development, where hyperparameters (e.g., learning rate) and/or feature selection can be tuned. Testing dataset should be entirely separate from training dataset.

*Validation:* Final verification of algorithm on another dataset. Ideally, this dataset is externally sourced from the datasets used for training and testing.

*Accuracy (ACC):* Percentage of observations in which model correctly predicts condition.

*Sensitivity (SENS):* Equivalent to true positive rate, i.e., percentage of observations in which model correctly predicts condition given that subject actually has the condition.

*Specificity (SPEC):* Equivalent to 1—false positive rate, i.e., percentage of observations in which model correctly predicts NO condition, given that subject does NOT actually have the condition.

*Precision (PREC):* Percentage of observations in which the subject actually has the condition, given the model predicted the subject had the condition, also known as positive predictive value.

*Area under the curve (AUC):* Calculated area under receiver operating characteristic (ROC) curve, which plots false positive rate (1—specificity) vs. true positive rate (sensitivity). Area of 1 indicates perfect model, area of 0.5 indicates model is equivalent to random guessing.

*F1 Score:* Measure of the harmonic mean between sensitivity and precision. Also known as the Dice similarity coefficient (DSC).

## Results

**Figure 1:**
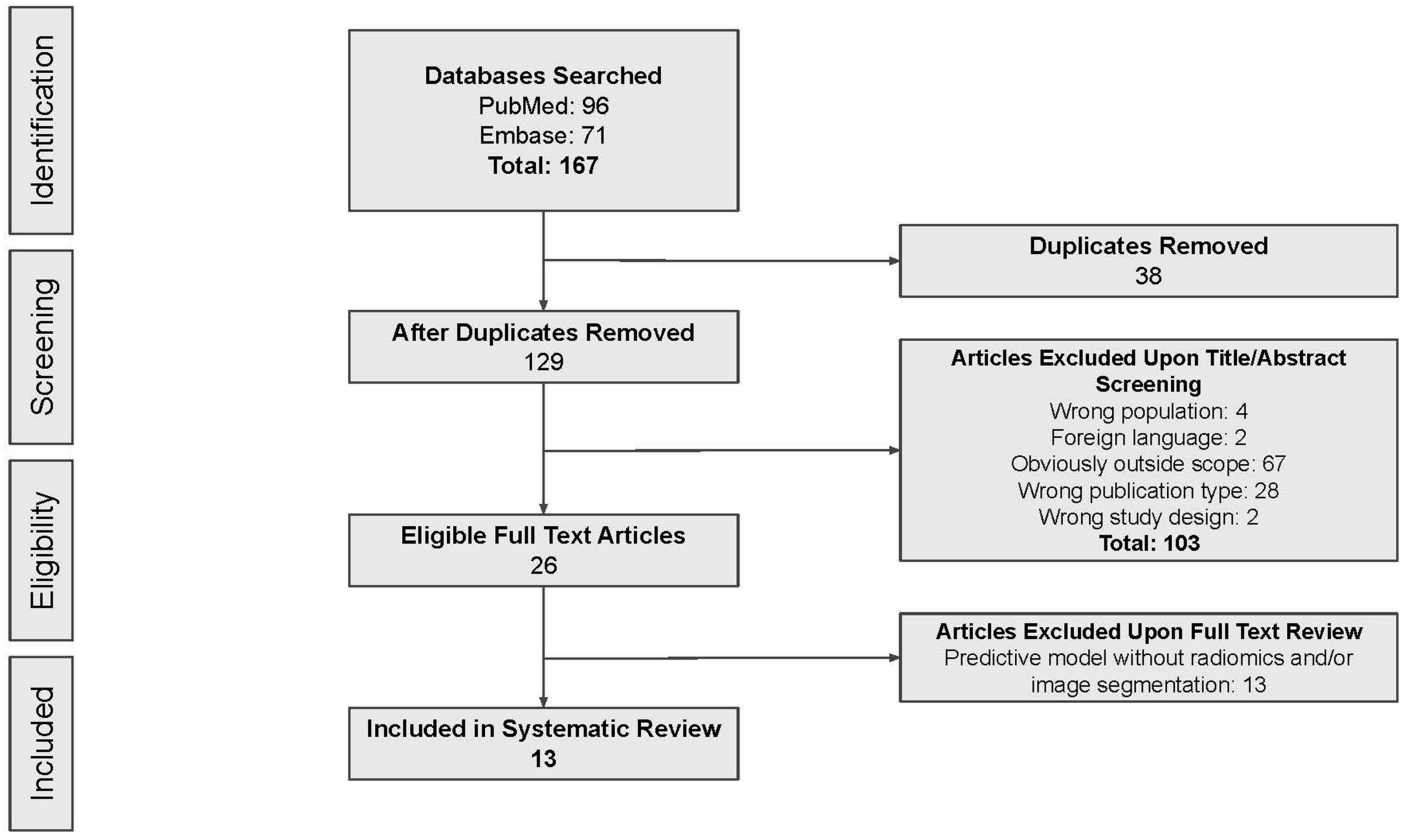
PRISMA flow diagram.

According to the search strategy described above, the search yielded a total of 167 articles. Of these, 13 studies were included in analysis with a total of 14 radiomic models described (Figure 1). Table 1 outlines the general study characteristics of each study. All of the included studies were retrospective in nature. Three of the studies (23%) dealt with AVM diagnosis and grading, 1 study (8%) gauged treatment response, 8 (62%) predicted outcomes, and the last one (8%) addressed prognosis. Table 2 reports the performance evaluation metrics of each study. Table 3 calculates the RQS for each study. The mean RQS was 15.92 (range: 10–18).

**Table 1:** Study characteristics.

	Study design	Objective(s)	Patients (N)	Imaging modalities	Scanner (Vendor)	AI Component	Segmentation method	Features (N)
Diagnosis and grading								
Zhu et al. 2023 (17)	Single-institution retrospective study	Evaluate hemodynamic differences in ruptured versus unruptured AVMs	529 (284 hemorrhage AVMs, 245 non-hemorrhage AVMs)	DSA	AXIOM-Artis (Siemens)	ML	Manual	5
Shi et al. 2021 (18)	Single-institution retrospective study	AVM diagnostic model AVM grading model	305 (153 normal and 152 AVMs)	DSA videos	UNIQ FD20 digital subtraction biplane angiographic system (Philips)	DL ML	NR	280
Zhang et al. 2019 (19)	Single-institution retrospective study	Diagnosis of AVM-related IPH vs. hematomas from another origin	261 (261 IPH)	NECT	Sensation 16 CT scanner (Siemens)	ML	Manual	11
Treatment response								
Meng et al. 2021 (20)	Single-institution retrospective study	Prediction of volume reduction velocity following fractionated SRS	30 (30 AVMs)	stereotactic MRI	1.5-T Magneton Vision (Siemens)	NR	Automated	13
Predictive outcomes								
Zhang et al. 2024 (21)	Single-institution retrospective study	Prediction of AVM rupture risk	896 (896 AVMs)	DSA	NR	ML	Manual	4
Jiao et al. 2023 (22)	Single-institution retrospective study	Prediction of motor deficits following AVM resection	83 (83 AVMs)	MRI	3.0-T Magnetom Trio (Siemens)	DL ML	Manual	4
Lin et al. 2023 (23)	Single-institution retrospective study	Prediction of seizures in unruptured AVMs	111 (111 unruptured AVMs)	T2-Weighted MRI	1.5-T Signa Cvi (GE Healthcare)	NR	Manual	73
Zhang et al. 2023 (24)	Multicenter retrospective study	Predicting rupture risk of AVMs	586 (368 hemorrhage AVMs, 218 non-hemorrhage AVMs)	CT	Revolution EVO (GE Healthcare)	ML	Manual	1790
Meng et al. 2022 (25)	Single-institution retrospective study	Predicting outcomes of SRS for AVM after partial embolization	130 (130 AVMs)	MRI	3.0-T Discovery MR 750 (GE Healthcare)	ML	Manual	4
Gao et al. 2022 (26)	Single-institution retrospective study	Predicting outcome of gamma knife radiosurgery for unruptured AVMs	88 (88 unruptured AVMs)	T1-weighted, T1 post-contrast, and T2-weighted MRI	3.0-T Discovery MR 750 (GE Healthcare)	ML	Manual	12
Zhao et al. 2021 (27)	Retrospective review of bAVM database of two prospective clinical trials (ClinicalTrials.gov Identifier: NCT01758211 and NCT02868008)	Prediction of epilepsy in unruptured AVMs	176 (176 unruptured AVMs; 100 w/ epilepsy and 76 w/o epilepsy)	TOF-MRA	3.0-T MR scanner (Siemens Trio)	ML	Manual	15
Zhang et al. 2019 (28)	Single-institution retrospective study	Prediction of epilepsy in unruptured AVMs	117 (117 AVMs)	T2-weighted MRIs	Magnetom Trio 3 T (Siemens)	ML	Manual	4
Prognosis								
Jiao et al. 2021 (29)	Retrospective review of bAVM database of two prospective clinical trials (ClinicalTrials.gov Identifier: NCT01758211 and NCT02868008)	Determination of AVM diffuseness	635 (635 AVMs)	TOF-MRA	3.0-T MR scanners (Siemens Trio, Philips Ingenia CX, and GE Discovery MR750)	ML	Automated	13

SRS, stereotactic radiosurgery; TOF, time of flight; MRA, magnetic resonance angiography; MRI, magnetic resonance imaging; CT, computed tomography; DSA, digital subtraction angiography; NR, not reported; DL, deep learning; ML, machine learning; NECT, Non-contrast enhanced CT; IPH, intraparenchymal hemorrhage.

**Table 2:** Model performance metrics.

Study	Training	Testing		Validation/final model
Zhu et al. 2023 (17)	AUC: 0.891 ACC: 0.863 SENS: 0.861 SPEC: 0.865 PPV: 0.877 NPV: 0.848	ACC: 0.849 SENS: 0.852 SPEC: 0.844 PPV: 0.881 NPV: 0.809		NR
Shi et al. 2021 (18)	**Diagnosis Model:** AUC: 0.942 ACC: 0.889 SENS: 0.943 SPEC: 0.823 **Grading Model:** AUC: 0.871 ACC: 0.840 SPEC: 0.866 SENS: 0.797	NR		**Diagnosis Model:** AUC: 0.971 ACC: 0.937 SENS: 0.911 SPEC: 0.967 **Grading Model:** NR
Zhang et al. 2019 (19)	AUC: 0.957 ACC: 0.926 SENS: 0.889 SPEC: 0.937 PPV: 0.800 NPV: 0.967	NR		AUC: 0.988
Meng et al. 2021 (20)	N/A	N/A		AUC: 0.83 SENS: 0.75 SPEC: 0.79
Zhang et al. 2024 (21)	AUC: 0.935 ACC: 0.869 SENS: 0.888 SPEC: 0.843	AUC: 0.933 ACC: 0.880 SENS: 0.914 SPEC: 0.831		AUC: 0.911 ACC: 0.840 SENS: 0.889 SPEC: 0.823
Zhao et al. 2021 (27)				
Jiao et al. 2023 (22)	NR	NR		AUC: 0.88 SENS: 0.92 SPEC: 0.74
Lin et al. 2023 (23)	NR	AUC: 0.817		NR
Zhang et al. 2023 (24)	**Random Forest** ROC: 0.982 ACC: 0.86 PREC: 0.99 F1 SCORE: 0.77	**Random Forest** ROC: 0.893 ACC: 0.79 PREC: 0.88 F1 SCORE: 0.64		ROC: 0.779
Meng et al. 2022 (25)	AUC: 0.78 ACC: 0.74	AUC: 0.77 ACC: 0.83		AUC: 0.78 ACC: 0.74
Gao et al. 2022 (26)	NR	NR		AUC: 0.88 ACC: 0.80 SENS: 0.92 SPEC: 0.60 PPV: 0.81 NPV: 0.82
Zhao et al. 2021 (27)	NR	NR		AUC: 0.82 SENS: 0.77 SPEC: 0.82 PPV: 0.84 NPV: 0.73 ACC: 0.78
Zhang et al. 2019 (28)	ACC: 0.822 AUC: 0.866	SENS: 0.786 SPEC: 0.769 ACC: 0.778		NR
Jiao et al. 2021 (29)	AUC: 0.93	**TEST #1** AUC: 0.95 ACC: 0.90 PREC: 0.81 RECALL: 0.84 F1 SCORE: 0.83	**TEST #2** AUC: 0.99 ACC: 0.95 PREC:0.84 RECALL: 0.94 F1 SCORE: 0.89	NR

NR, Not Reported; N/A, Not Applicable; AUC, area under the receiver operating characteristic curve; ACC, accuracy; SENS, sensitivity; SPEC, specificity; PPV, positive predictive value; NPV, negative predictive value; PREC, precision; ROC, receiver operating characteristic curve.

**Table 3:** Calculation of radiomics quality score (RQS).

Variable (maximum points)	Shi et al. 2021 (18)		Zhang et al. 2019 (19)	Meng et al. 2021 (20)	Zhao et al. 2021 (27)	Jiao et al. 2021 (29)	Zhang et al. 2019 (28)
	Diagnosis	Grading					
Image protocol quality (2 points)	+1	+1	+1	0	+1	+1	+1
Multiple segmentation (1 point)	0	0	+1	0	+1	+1	+1
Phantom study (1 point)	0	0	0	0	0	0	0
Imaging at multiple time points (1 point)	+1	+1	0	0	0	0	0
Feature reduction or adjustment for multiple testing (3 points)	+3	+3	+3	+3	+3	+3	+3
Multivariable analysis (1 point)	0	0	+1	0	+1	+1	+1
Biological correlates (1 point)	0	0	0	0	0	0	0
Cut-off analysis (1 point)	+1	+1	+1	+1	+1	+1	+1
Discrimination statistics (2 points)	+2	+2	+2	+1	+2	+2	+2
Calibration statistics (2 points)	0	0	0	0	+2	0	0
Prospective study (7 points)	0	0	0	0	0	0	0
Validation (5 points)	+2	+2	+2	0	+2	+2	+2
Comparison to ‘gold standard’ (2 points)	+2	+2	+2	+2	+2	+2	+2
Potential clinical applications (2 points)	+2	+2	+2	+2	+2	+2	+2
Cost-effectiveness analysis (1 point)	0	0	0	0	0	0	0
Open science and data (4 points)	+1	+1	0	+1	+1	+1	0
Total: 36	**15**	**15**	**15**	**10**	**18**	**16**	**15**
Variable (maximum points)	Zhang et al. 2024 (21)	Lin et al. 2023 (23)	Gao et al. 2022 (26)	Zhang et al. 2023 (24)	Jiao et al. 2023 (22)	Zhu et al. 2023 (17)	Meng et al. 2022 (25)
Image protocol quality (2 points)	+1	+1	+2	+1	+1	+1	+1
Multiple segmentation (1 point)	0	0	+1	+1	+1	+1	0
Phantom study (1 point)	0	0	0	0	0	0	0
Imaging at multiple time points (1 point)	0	0	0	0	0	0	0
Feature reduction or adjustment for multiple testing (3 points)	+3	+3	+3	+3	+3	+3	+3
Multivariable analysis (1 point)	+1	+1	+1	+1	+1	+1	0
Biological correlates (1 point)	0	0	0	0	0	0	0
Cut-off analysis (1 point)	+1	+1	0	+1	+1	+1	+1
Discrimination statistics (2 points)	+2	+2	+2	+2	+2	+2	+2
Calibration statistics (2 points)	0	+2	+1	+1	+1	0	0
Prospective study (7 points)	0	0	0	0	0	0	0
Validation (5 points)	+2	0	+2	+3	+2	+2	+2
Comparison to ‘gold standard’ (2 points)	+2	+2	+2	+2	0	+2	+2
Potential clinical applications (2 points)	+2	+2	+2	+2	+2	+2	+2
Cost-effectiveness analysis (1 point)	0	0	0	0	0	0	0
Open science and data (4 points)	0	0	0	0	0	0	0
Total: 36	**14**	**14**	**16**	**17**	**14**	**15**	**13**

## Discussion

**Figure 2:**
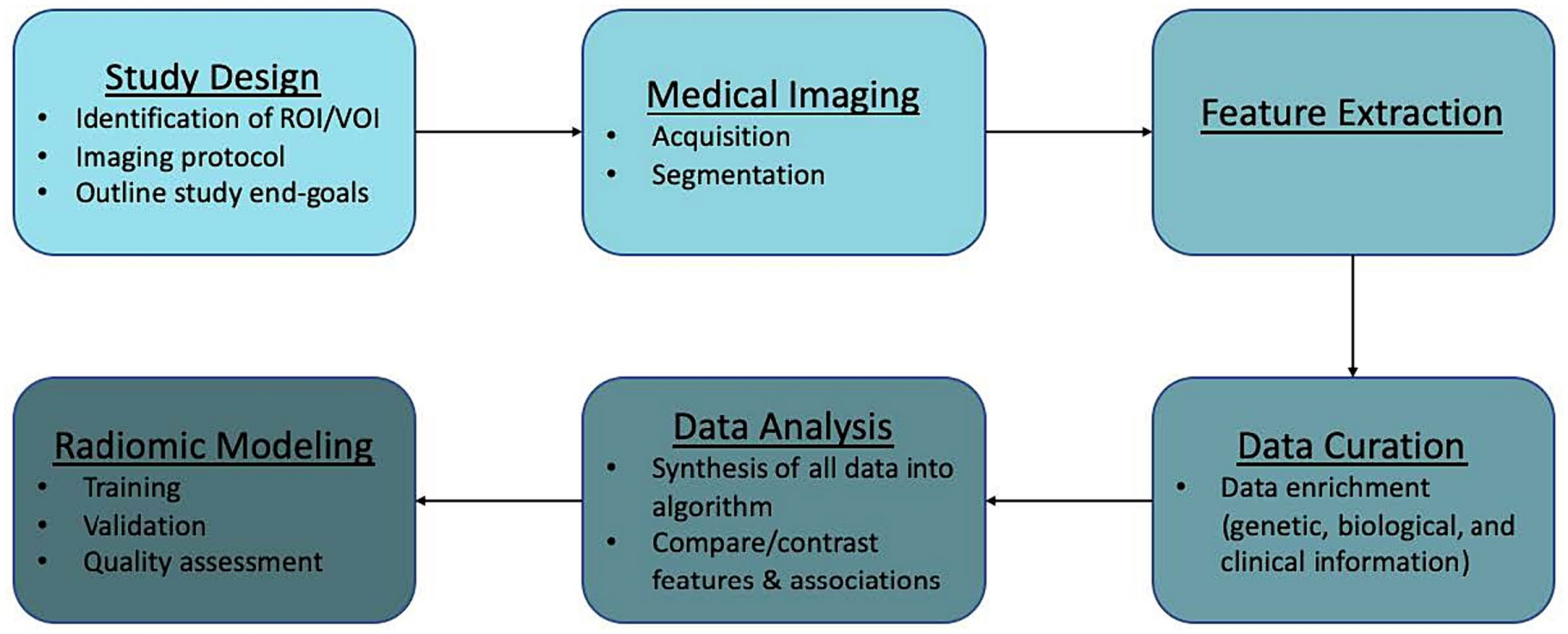
Radiomics workflow.

Recent studies have examined the efficacy of radiomics in AVM diagnosis, prediction of epilepsy in unruptured AVMs, and cerebrovascular anatomical mapping for SRS planning, among others. Here, we discuss the workflow of radiomics in AVMs (Figure 2) along with its potential applications in routine use, limitations, and future directions.

### Radiomics workflow

Radiomics analysis can be applied to virtually any image generated in a clinical setting. Workflow is generally divided into the following components: study design and planning, medical imaging, feature extraction, data curation, data analysis, and modeling. We outline this general workflow and provide caveats to be mindful of when dealing with AVMs.

### Study design and planning

The first step in radiomics analysis is the identification of a clinical hypothesis. After this, the appropriate region of interest (ROI) or volume of interest (VOI) and analysis end-points can be identified. For our purposes, the ROI/VOI would be the patient’s AVM. However, it is also possible to perform radiomic analyses on normal tissue or specific sections of the lesion. In these cases, study of the feeding arteries, intranidal features, or draining veins may be appropriate. Also, perfusion data to evaluate phenomena like steal or normal perfusion pressure breakthrough might be useful.

### Medical imaging

#### Image acquisition

An advantage to the implementation of radiomics in AVMs is that it utilizes routine clinical images obtained in patient management such as CT, CTA, MRI, MRA, and DSA. An imaging protocol is put into place where medical images are obtained from large, established patient databases or ones that are newly created. Ideally, imaging protocols would be described in-depth for reported models. There are currently no universal standardized imaging protocols. Standardizing imaging protocols and/or disclosing current protocols utilized is necessary in order to provide reproducible and comparable results across studies (16). Imaging patients multiple times can also mitigate the variability in radiomics features seen with motion degradation (16, 30).

#### Lesion segmentation

Once identified and saved within a database, images are stored as their essential components. Segmentation of the ROI/VOI can then be performed manually or with auto-segmentation, which involves the use of automatic or semiautomatic algorithms. A number of studies have been described which utilize machine learning for AVM segmentation in both MR imaging and CT (28, 31, 32). Dice Similarity Coefficient (DSC) is one of the most commonly used performance metrics in the imaging segmentation literature. Unfortunately, this metric is not yet uniformly associated with clinical utility (3, 33). During the process of segmentation, the choice of which voxels, a unit of graphical information that defines a three dimensional space, to incorporate into analysis is determined. This is a crucial step as production of different radiomic features can result from different segmentation techniques (manual vs. semi-automatic vs. automatic) and leads to bias during analysis (16). In order to improve quality of the model, and limit bias, multiple segmentations by different algorithms or physicians can be performed (16).

#### Feature extraction

Qualitative and quantitative information can be derived from medical images. Qualitative features are what is commonly used in radiological vernacular. Today, countless quantitative features can be identified and extracted using high-throughput computing. Feature extraction involves the use of manual image processing or AI to extract a large number of features from medical imaging via mathematical algorithms. After segmentation, individual features can be assessed for correlation to a chosen outcome. Extracted quantitative features can be loosely broken into three categories: shape, signal distribution, and texture (34). Table 4 further categorizes these subgroups. Any combination of these features can lead to the generation of hundreds of variables from a single image (34).

**Table 4:** Radiomic quantitative features.

Feature	Definition	Examples
Shape features	Geometric properties of the ROI/VOI	Shape, volume, orthogonal direction maximum diameter, surface area, compactness, surface-to-volume ratio
First-order statistics features	Distribution of the image voxel values	Voxel intensity mean, median, maximum, minimum; asymmetry, flatness, uniformity, entropy
Second-order statistics features (aka textural features)	Calculation of the relationships between neighboring voxels via statistical methods	Spatial arrangement, intra-lesion heterogeneity
Higher-order statistics features	Features obtained via statistical methods after the application of filters or mathematical algorithms	Identification of repetitive or non-repetitive patterns, noise suppression, highlighting details

One important variable to consider during feature extraction is the scanner type and vendor. Studies which utilize various scanners must take this into account as it can affect subsequent analyses and may not be externally valid (30). One method to address the uncertainty seen between scanners and vendors is the implementation of phantom studies. These studies use a specifically engineered object which provides an optimal imaging scenario to evaluate and tune performance among different imaging devices (16, 30).

#### Data curation

This part of the workflow deals with quality analysis and is often the limiting stage. At this point, radiologic data can be enriched with the addition of other patient data including genetic, biological, and clinical information.

#### Data analysis

During analysis, all of the information incorporated into the algorithm (radiologic, genetic, biological, clinical) is synthesized and repetitive components are removed. Then, features can be compared and contrasted in order to examine specific outcomes.

#### Construction of radiomics models

Radiomics has been mostly employed in tumor pathology with few studies involving vascular pathology. Current AVM radiomics models have yet to undergo external validation and are not ready for clinical use. Key components of modeling include training, validation, and quality assessment.

#### Training and validation

Machine learning-based radiomics models must undergo training and validation before clinical use. Performance metrics are most commonly reported as discrimination statistics such as ROC, AUC, accuracy, sensitivity, specificity. There is heterogeneity in how these metrics are reported. Specifically, studies do not uniformly report statistical methods for both training and validation models. Training and validation models should demonstrate consistency in reported statistics (16). There is also confusion in commonly used terminology. Protocols for reporting guidelines in radiomics models and their validation process could lead to improved quality of these studies (14). Lambin et al. (16) described how to perform quality assessment of radiomics models at length. The mean RQS of our studies was 15.92 (range: 10–18). This is comparable to the values of radiomics models in other fields (35–41).

### Clinical applications of radiomics in AVMs

#### Diagnosis and grading

Shi et al. (18) described a hybrid machine learning/deep learning network which utilized temporal and spatial features from DSA videos to diagnose and grade AVMs. This was one of the few studies which studied DSA videos—arguably the most important imaging technique in evaluation of cerebrovascular diseases (42). While some would argue that the diagnosis of AVM is clear without utilizing advanced models, this is not always true in inexperienced centers. Particularly in emergency situations, a tool which aids in diagnosis could lead to faster treatment.

An advantage to using DSA in diagnosis is that it can provide in-depth detail about the cerebrovascular anatomy, including specific arteries feeding the AVM, venous drainage pattern, and perfusion and distribution patterns. Such advantages are highlighted in Zhu et al.’s work,32 which used machine learning analysis of DSA to evaluate hemodynamic differences in ruptured versus unruptured AVMs. This is an advantage over models of AVM diagnosis which utilize 4D-CTA and 3D-RA but do not allow for visualization of vascular perfusion and distribution within the AVM (18, 43–45). Extraction of relevant data from DSA videos had not been proposed prior to the work done by Shi et al. (18) In order to do so, they created a hybrid machine/deep learning model which extracted information from DSA videos by using automated methods to collect information on AVM perfusion and distribution and then combining that with information gleaned from intensity, texture, and wavelet radiomic features taken from static images.

Zhang et al. (19) produced a successful radiomics model for differentiating between AVM-related hematomas versus hematomas from a different etiology using NECT. This is a clinically useful tool as NCET is often the first step in evaluation of suspected intracranial hemorrhage. Distinguishing AVM-related hematomas by the naked eye remains difficult and angiography remains the gold standard. However, angiography can be time consuming and requires the use of contrast agents and patient cooperation. Zhang et al. described their model as a fast, non-invasive method which did not require the use of contrast agents for diagnosis. Their model demonstrated that hematomas resulting from AVM had a larger diameter, coarser texture, and more heterogeneous composition when compared to non-AVM hematomas (19). Their model also outperformed interventional radiologists when comparing measures of specificity and accuracy (19). Distinguishing between AVM-related hematoma and hematomas from other etiologies allows for a faster, more accurate treatment response with targeted utilization of resources.

#### Treatment planning

A significant portion of the work involving ML and DL for AVM has been devoted to methods of delineating the AVM nidus and surrounding structures in preparation for radiosurgery. Specifically for describing methods of auto-segmentation of the ROI/VOI for precise SRS target planning (3, 31, 46). While these studies do not fit the criteria for radiomic modeling, they warrant discussion as they show promise for future studies.

Simon et al. (3) created the first model attempting to generate cerebrovascular anatomical maps in AVM patients. They provided a proof-of-principle study using multiple MRI sequences demonstrating an effective method for producing high-resolution images capable of differentiating not only the AVM nidus, but collateral vessels and surrounding parenchyma for pre-SRS planning. Their model was also able to differentiate those vessels that had been previously embolized. Wang et al. (31) also successfully described this method using CT.

#### Treatment response

It is currently unclear which AVM patients, particularly those with large or high-grade AVMs, could potentially benefit from DS-SRS (dose-staged SRS) versus *VS*-SRS (volume-staged SRS). A benefit for DS-SRS is that it allows for multiple sessions of targeted radiation as opposed to a single, large dose (47). Meng et al. (20) extracted radiomic features from stereotactic MR images in order to predict the rate of nidus obliteration (volume reduction velocity) following DS-SRS (dose-stage SRS). They found one radiomic feature (SurfaceVolumeRatio) that was associated with volume reduction velocity. Following DS-SRS, a smaller SurfaceVolumeRatio predicted a higher volume reduction rate. This supported the author’s statement that DS-SRS is more appropriate for AVM niduses which are large and compact as AVM obliteration may be achieved faster (20).

#### Predictive outcomes and prognosis

Three of the included studies predicted epilepsy in unruptured AVMs (23, 27, 28). Zhao et al. (27) retrospectively reviewed the database of 2 prospective clinical trials in order to accurately predict epilepsy from TOF-MRAs. Zhang et al. (28) and Lin et al. (23) produced similar results with T2-weighted MR imaging. Early prediction of seizure development in unruptured AVMs could lead to a change in management advocating for more aggressive intervention.

To date, two radiomics models have been reported that assess the risk of rupture in AVM patients, utilizing DSA and CT imaging (21, 24). In young adults, AVMs are one of the most common causes of intracranial hemorrhage (ICH) resulting in a morbidity rate of 30–50% and a mortality rate of 10–15% (18, 48). While the yearly rupture rate of AVMs is about 2–4%, prognostic factors that alter this risk are uncertain.

AVM diffuseness is a prognostic factor of microsurgical resection and has been associated with hemorrhagic risk (29). However, this value can sometimes be hard to quantify and exist along a spectrum. Jiao et al. (29) developed a machine learning algorithm that was capable of determining diffuseness from extracted first-order statistical radiomic features on 3D TOF-MRA. This could help stratify patients before surgical intervention.

Other ML approaches have been described in the prognosis of AVM management. Following SRS, it can take up to 3 years for an AVM to be fully obliterated (46). In this time period, it is important for these patients to remain under clinical surveillance for brain edema and other radiation effects. Meng et al. (25) and Gao et al. (26) described radiomics models utilizing MRI for predicting outcomes of SRS after partial embolization and gamma knife radiosurgery, respectively. Moreover, although not in the realm of radiomics, Yang et al. (32) and Peng et al. (46) both described ML algorithms that were capable of predicting radiation-induced changes following SRS. Using T2-weighted MR images, Yang et al. (32) calculated the volume and proportion of brain parenchyma within the 12 Gy radiosurgical volume (V12) in patients with unruptured AVMs and previous SRS. They found that the volume of brain parenchyma within the 12 Gy radiosurgical volume (V12) following SRS was associated with both early and late radiation-induced changes (32). Similarly, Peng et al. (46) developed an algorithm which was capable of automatically segmenting different regions within the prescription isodense region. This could predict adverse effects in the latency period. Interestingly, radiomics analyses may also target specific outcomes, as Jiao et al. (22) described deep and machine learning methods for predicting motor deficits following AVM resection. These findings underscore the utility of radiomics in postoperative care.

### Future directions

Radiomics in AVM management is still in its infancy. However, the described radiomics models in our analysis show great promise for the future.

#### Precision medicine

In the era of precision medicine, radiomics has the potential to tailor AVM management as individual patient data can be converted to digital images and mineable data. It is feasible that radiomics will become routine practice in the future as these analyses are intended to be conducted with standard of care medical images (12). Eventually, it is possible that radiomics can personalize AVM management by selecting the most appropriate therapy for each patient and identify more rapidly if it is not working. An area of particular interest would be the use of radiomics to guide treatment of high-grade AVMs, which currently involves considerable debate (49). Radiomics could also assist in the assessment of AVM genetics. The combination of radiological data and genetic data is known as radiogenomics. AVMs represent an interesting pathology for implementation of radiogenomics given recent findings in genetic biomarkers.

Specific to AVMs, the ability of radiomics to handle large volumes of data could lead to both better predictive models describing the natural history as well as treatment response and success. For instance, utilizing quantitative magnetic resonance angiography, flow rates in the feeding arteries and draining veins could be calculated. At this time, it is unknown if these measurements are predictive of rupture or predict a positive or negative treatment outcome (50–52). Due to the large amount of data, it is difficult to determine these relationships manually. Using radiomics, ML and AI could easily be used to study these parameters over a much larger data set.

#### Artificial intelligence in AVM management and other cerebrovascular diseases

A large part of the growing field of radiomics is the integration of AI methods into models. It is important to note that the goal of AI is not to replace physicians; rather it acts as a clinical tool. One study showed that a combined model incorporating both a clinical and radiomics model performed better than either model alone (27). In its most basic form, AI is any technology that uses explicit programming codes in an attempt to mimic human behavior. The fact that a code would have to be written for any and every given scenario, makes it rudimentary and non-ideal for AVM management as many thousands of variables could be present given an individual’s anatomy and past medical history. Machine learning (ML) is a subset of AI. It is more sophisticated as it does not rely on explicit programming. Instead, the machine is able to learn the programming rules. However, it is not a fully intelligent method as humans are still defining the features of the programming. One advantage of AI in radiomics studies is the elimination of bias. Evaluation of these films by the operating surgeon or radiation oncologist often introduces bias as that physician already may have a preconceived notion of what they would like to do. Similarly, they may be biased toward one treatment outcome or another. Radiomics would allow the use of objective data to evaluate treatment outcomes and surgical risk, eliminating this type of bias.

Deep learning (DL) is a subset of ML inspired by the brain’s biological neural networks. This technology has the capability to create its own neural networks. When this neural network has multiple layers, it is called a deep neural network. A recent meta-analysis examining DL in neurosurgery showed promising results of DL models (53). However, their review demonstrated that aside from public databases, there was a paucity of readily available data (53). Especially when dealing with large data stores, large-scale data sharing is necessary to promote transparency, create reproducible results, and mitigate bias (53).

### Study limitations

Limitations of the included studies pertain to the rarity of AVMs. This is overcome, in part, by the large amount of data that can be extracted from a single image. While ML and DL systems require hundreds to thousands of training examples before outputting predictions, MRIs contain hundreds of slices so the imaging from a relatively small number of patients is adequate (3). Nonetheless, validating these models still requires an ample set of AVM patients. There is also a heterogeneous nature to the pathology and patients differ considerably in prior symptom (e.g., seizure, hemorrhage) and treatment course (e.g., prior microsurgery, SRS, embolization). Detailed patient demographics can help subclassify these patients in the future. Many of the current models also have to exclude patients with other neurological diseases such as moyamoya and brain tumors, which could lead to bias during validation of the model.

Limitations of our analysis include a small amount of included studies. The limited number of studies and heterogeneity of current literature precluded us from performing a meta-analysis of the included studies. Additionally, the RQS score was originally created for radiomics within oncology. However, it is a widely applicable assessment for radiomic models. In the future, a quality assessment model for radiomic models specific to AVM may be suggested.

## Conclusion

The authors’ results demonstrated that radiomics is currently being studied in different facets of AVM management. Additionally, other ML/DL studies are paving the way for other predictive models. More prospective studies are warranted to determine the role of radiomics in the diagnosis, prediction of comorbidities, and treatment selection in AVM management. Further collaborative efforts between neurosurgeons, radiologists, biomedical engineers, and data scientists has the potential to result in algorithms that successfully assess AVMs and individualize patient management.

## Data availability statement

The original contributions presented in the study are included in the article/supplementary material, further inquiries can be directed to the corresponding author.

## Author contributions

AG: Writing – review & editing, Writing – original draft, Data curation, Conceptualization. AE: Data curation, Writing - review & editing. GE: Writing – review & editing, Data curation, Conceptualization. CB: Writing – review & editing, Formal analysis, Data curation. XZ: Writing – review & editing. AB: Supervision, Writing – review & editing, Conceptualization.
